# Mechanism of Danggui Sini underlying the treatment of peripheral nerve injury based on network pharmacology and molecular docking: A review

**DOI:** 10.1097/MD.0000000000033528

**Published:** 2023-05-12

**Authors:** Ning Zhang, Dandan Zhang, Qian Zhang, Ruisu Zhang, Yan Wang

**Affiliations:** a Heilongjiang University of Chinese Medicine, Harbin, China; b Dalian Port Hospital, Dalian, China; c The Second Affiliated Hospital of Heilongjiang University of Chinese Medicine, Harbin, China; d Dalian Women and Children’s MedicalGroup, Dalian, China; e The First Affiliated Hospital of Heilongjiang University of Chinese Medicine, Harbin, China.

**Keywords:** Danggui Sini, molecular docking, network pharmacology, peripheral nerve injury, TCM

## Abstract

Danggui Sini is a traditional Chinese medicine prescription for treating peripheral nerve injury (PNI). We studied the mechanisms of this decoction through network pharmacology analysis and molecular docking. Using R language and Perl software, the active components and predicted targets of Danggui Sini, as well as the related gene targets of PNI, were mined through TCMSP, GeneCards, OMIM, TTD, and DrugBank. The network diagram of active components and intersection targets was constructed using Cytoscape software and the STRING database. The CytoNCA plug-in was used to screen out the core compounds and key targets. The genes were analyzed for Gene Ontology and Kyoto Encyclopedia of Genes and Genomes enrichment. AutoDock was used to analyze the molecular docking of key targets and core compounds of diseases. The drug component disease target regulatory network showed that the key components included quercetin, kaempferol, naringenin, and licochalcone A, which play key roles in the whole network and may be the primary compounds associated with the action of Danggui Sini against PNI. PPI network topology analysis showed high degree values for RELA, JUN, MAPK1, RB1, and FOS. Enrichment analysis showed that the core targets of Danggui Sini participated in pathways associated with neurogenesis-multiple diseases. Molecular docking showed that the active ingredients in Danggui Sini had a good binding ability with key targets. We conclude that many active components of Danggui Sini play therapeutic roles in PNI treatment by regulating RELA, JUN, MAPK1, RB1, and FOS, and multiple other targets in inflammation, immunity, and lipid metabolism.

## 1. Introduction

Peripheral nerve injury (PNI) is a serious disease that leads to partial functional impairment and permanent disability of the segmental motor and sensory and autonomic nerves. Most peripheral nerve injuries require surgical repair so that axons can regenerate and attach to the distal end of the nerve. Functional recovery following surgery is limited by the size of the nerve space, neuroma, and scar tissue formation.^[1]^ Therefore, neural function is slow to recover, incomplete, and accompanied by pain.^[2]^ During the delay in postoperative regeneration, nerve mismatch leads to further delays in functional repair for several days to several months.^[3–5]^ Only 40% to 50% of patients who received autologous nerve transplantation recovered sufficient functionality.^[6,7]^ This delay in functional recovery indicates the need for drug intervention. Therefore, new drug treatment strategies are needed to promote the functional recovery of patients with PNI.

Chinese herbal medicine is rich in resources and has been used for thousands of years, providing a promising means to treat PNI. Traditional Chinese Medicine (TCM) prescriptions comprise a variety of compounds with different structures and functions that act on multiple targets for multi-level treatment.^[5]^ The Danggui Sini decoction^[8]^ is a prescription from the Treatise on Febrile Diseases composed of *Angelica sinensis* (Danggui, DG), Ramulus Cinnamomi (Guizhi, GZ), asarum (Xixin, XX), tongcao (TC), peony (Shaoyao, SY), jujube (Dazao, DZ), and *Glycyrrhiza uralensis* (Gancao, GC). *A. sinensi*s and cinnamon twig are the primary drugs in the prescription. Asarum and peony are official medicines. Tongcao, jujube, and *G. uralensis* are adjuvants. The whole prescription warms meridians, disperses cold, nourishes the blood, and dredges the pulse. Modern research has demonstrated the pharmacological effects of *A. sinensis*, such as hematopoiesis, antioxidation, anticancer, neuroprotection, and immune regulation, and indicated its use for the treatment of nervous system diseases.^[9,10]^ Ramulus Cinnamomi (volatile oil) has many pharmacological effects, such as anti-inflammatory, anti-tumor, antibacterial, anti-diabetes, anti-obesity, and neuroprotection and can also be used for the treatment of nervous system diseases.^[11,12]^ Peony has antioxidant, anti-inflammatory, anti-tumor, antibacterial, anti-virus, cardiovascular protection, and neuroprotective properties.^[13]^ Asarum has analgesic, anti-inflammatory, neuroprotective, cardiovascular protection, antitussive, immunosuppressive, antitumor, and microbicide effects.^[14]^ The pharmacological effects of *G. uralensis* are anti-inflammatory, antioxidant, anti-allergic, and antibacterial.^[15]^ Tongcao has anti-inflammatory and other biological properties.^[16]^ Jujube elicits anti-cancer, anti-oxidation, anti-inflammatory, anti-hyperlipidemia, anti-hyperglycemia, immune regulation, neuroprotection, sedation, and anti-virus effects.^[17]^ Cheng et al^[18]^ found that the Danggui Sini decoction can significantly decrease the expression levels of NF-κB and mRNA, which play protective roles in nerve cells. However, the mechanism of the Danggui Sini decoction in the treatment of PNI remains unclear owing toa lack of systematic analysis.

The scope of network pharmacology is expanding, and current research has aimed to clarify the effective components and therapeutic activities of TCM.^[19]^ Systematically analyzing the multi-component and multi-target mechanisms of TCM at the network level, its holistic view, and systems theory are consistent with the holistic concept of TCM and the principles of multi-target and multi-component TCM prescriptions. It is an effective technical method to study the compatibility and pharmacological effects of TCM.^[20,21]^ Molecular docking technology can improve pharmacological experiments by allowing the theoretical simulation of interactions between receptors and ligands and predicting their binding mode and affinity. Both have been widely used in TCM to explore treatment mechanisms associated with diseases that affect the nervous, circulatory, and respiratory systems; diabetes; cancer; and osteoarthropathy.^[22–27]^

**Figure 1.**
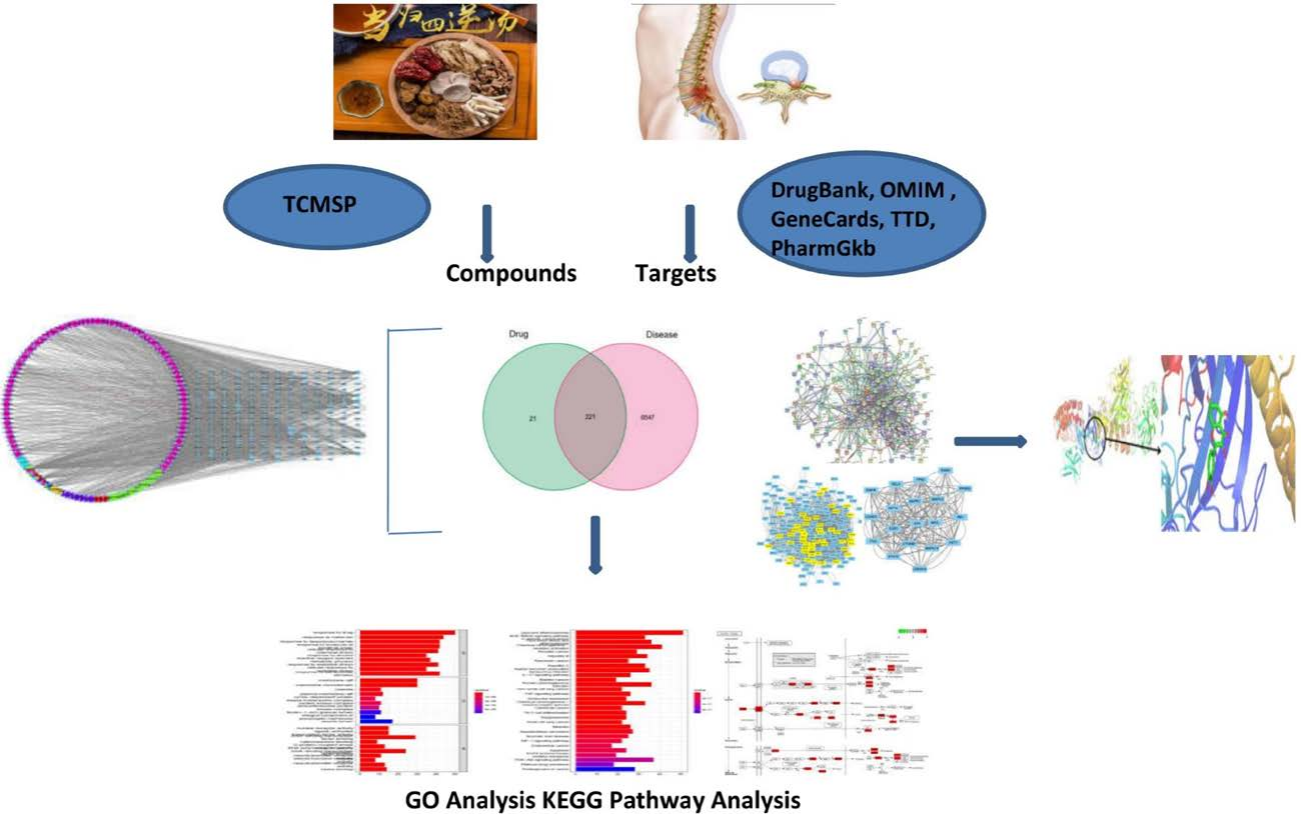
Overall flow chart of this study. GO = gene ontology, KEGG = Kyoto Encyclopedia of Genes and Genomes, OMIM = Online Mendelian Inheritance in Man, TCMSP = Traditional Chinese Medicine Systems Pharmacology, TTD = Therapeutic Target Database.

This study aimed to predict the targets of the Danggui Sini decoction through constructing protein–protein interaction (PPI) networks, screening topologies, and performing molecular docking analysis. Our results revealed the mechanism of Danggui Sini decoction in the treatment of PNI and provide a basis for future pharmacological experiments and clinical application. The overall flow chart of this study is shown in Figure 1.

## 2. Materials and methods

### 2.1. Screening of active components and target proteins

In the Traditional Chinese Medicine Systems Pharmacology (TCMSP) database (https://tcmsp-e.com/), *A. sinensis*, Ramulus Cinnamomi, asarum, tongcao, peony, jujube, and licorice were used as keywords to search for the active ingredients. Oral bioavailability (OB) ≥ 30% and drug like (DL) ≥ 0.18 were used to screen for the target active ingredient group. Then, we matched the active ingredients with potential targets one by one according to the TCMSP database using UniProt (https://uniprot.org/). The target was standardized to “Homo sapiens” as the target of the active ingredient. Using Perl (https://www.perl.org/),the gene targets of the effective components of the Danggui Sini decoction were obtained.

### 2.2. PNI disease target screening

In the Therapeutic Target Database (TTD) (https://db.idrblab.net/ttd/), DrugBank (https://www.drugbank.com), GeneCards (https://www.genecards.org), Online Mendelian Inheritance in Man (OMIM) (https://www.omim.org), and PharmGKB (https://www.Pharmgkb.org) database, we used the keyword “peripheral nerve injury” to retrieve disease targets. Using R (Venn diagram package), we merged disease-related genes, removed duplicate targets, and integrated PNI-related targets.

### 2.3. Construction of target PPI network

Using R and the Venn diagram package, the gene targets of the effective components were analyzed to determine the target of the Danggui Sini decoction for the treatment of PNI. Cytoscape 3.8.2 was used to construct the network diagram of active ingredients and intersection targets. We then input the intersection target into STRING (https://string-db.org/); the species was set to “homo sapiens,” the lowest interaction threshold was set to the highest confidence (0.9), and the PPI network information was obtained. Then, topology analysis of the PPI network was carried out using the CytoNCA plug-in in Cytoscape 3.8.2, including the betweenness centrality (BC), degree centrality (DC), closeness centrality (CC), and centrality of the eigenvector (EC). The local average connectivity (LAC) and network centrality (NC) scoring conditions were filtered twice. For screening, the value corresponding to the median node was calculated. Nodes higher than this value were defined as representing significant effective compounds and key targets.

### 2.4. GO enrichment analysis and KEGG pathway analysis

Using R packagesdose, clusterprofiler, and enrichlot, we carried out Gene Ontology (GO) analysis of biological processes, cell composition, and molecular functions and Kyoto Encyclopedia of Genes and Genomes (KEGG) pathway enrichment analysis. The significance threshold was *P* < .05. We also drew histograms to further illustrate the mechanism of the Danggui Sini decoction in the treatment of PNI.

### 2.5. Molecular docking

The mol format of the 2D structure of small molecule ligands was downloaded from the PubChem database (https://www.ncbi.nlm.nih.gov/geo/), and chemistry software was used to convert the 2D structure into a 3D structure. From the RCSB Protein Data Bank (PDB) database (http://www.rcsb.org/), we downloaded the 3D structure PDB file of the core target protein, used PyMOL to remove water molecules and small molecule ligand molecules, used autodock to dock molecules based on human protein, and used Vina and PyMOL to plot the results with the lowest binding energy between each target and pharmacodynamic components.

### 2.6. Ethics and dissemination

Ethical approval is not required for this network pharmacology and molecular docking analysis as we did not use data related to individual patient. The final report of this paper will be published in a peer-reviewed scientific journal or at conferences to provide evidence-based medical support on PNI, its genetics, molecular pathogenesis and new therapeutic targets for clinical workers, and dataset will be made freely available.

## 3. Results

### 3.1. Prediction of active components and related targets

From the TCMSP database, taking OB ≥ 30% and DL ≥ 0.18 as the screening conditions, 155 active ingredients were screened, including 2 *A. sinensis*, 7 Ramulus Cinnamomi, 8 asarum, 4 thoroughfare, 13 peony, 29 jujube, and 92 *G. uralensis*. Furthermore, 2656 activity targets were determined, and 242 active ingredient-related targets were determined after eliminating duplicate targets.

### 3.2. Prediction of PNI disease targets and acquisition of intersection targets

**Figure 2.**
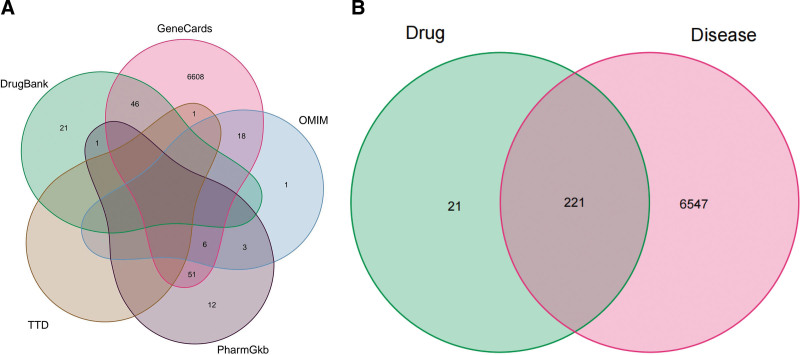
Venn diagrams of (A) PNI target and (B) Sini decoction and PNI target. OMIM = Online Mendelian Inheritance in Man, PNI = peripheral nerve injury, TTD = Therapeutic Target Database.

A total of 6768 disease-related targets were determined by searching the TTD, DrugBank, GeneCards, OMIM, and PharmGKB databases (Fig. 2A). Venn analysis was conducted on the gene targets of the active components and PNI disease targets, and we determined 221 targets for PNI (Fig. 2B).

### 3.3. Components and disease target regulatory network

**Figure 3.**
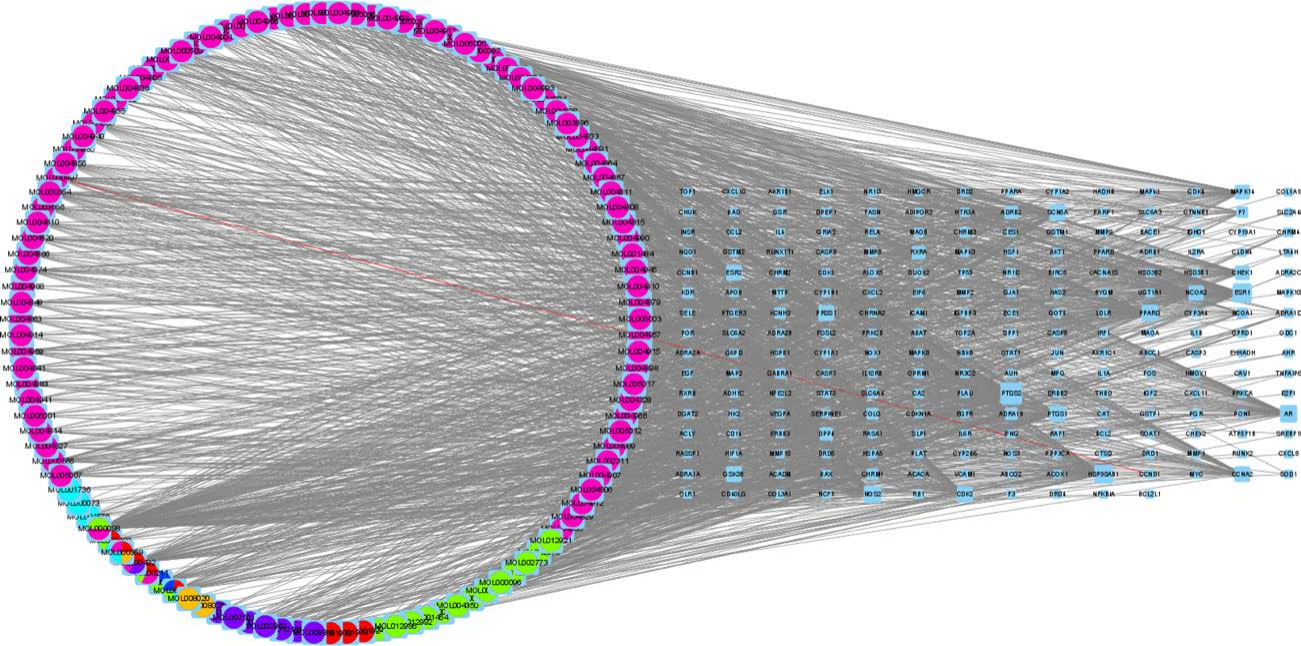
Danggui Sini Decoction–component–disease regulation network diagram.

Cytoscape 3.8.2 was used to establish the interaction network between active ingredients and targets. The nodes represent TCM, active ingredients, and targets, and the edges represent the interaction between active ingredients and target diseases. The network contained 340 nodes and 1729 edges (Fig. 3). The left circle represents the effective ingredients, and the different colors represent the different drugs that the ingredients were derived from (pink, *G. uralensis*; red, peony; green, jujube; purple, asarum; orange, tongcao; blue, Ramulus Cinnamomi; multi-colored, different drugs). The grid on the right represents the target genes for the treatment of PNI (expressed in blue). The larger the square area in the grid, the more components connected with the gene. The top 9 active components in the target are quercetin, isoflavone, glabridin, kaempferol, naringenin, formononetin, licochalcone A, beta sitosterol, and stigmasterol, which can interact with 143, 108, 67, 49, 33, 29, 28, 28, and 26 target proteins, respectively. Regarding targets, 11.31% of the targets interact with 20 or more compounds, and20% interact with ≥ 30 compounds. The top 7 targets are PTGS2, ESR1, HSP90AB1, AR, NOS2, PPARG, and SCN5A, which can interact with 103, 82, 77, 68, 67, 64, and 57 compounds, respectively.

### 3.4. Construction and network topology analysis and PNI PPI network

**Figure 4.**
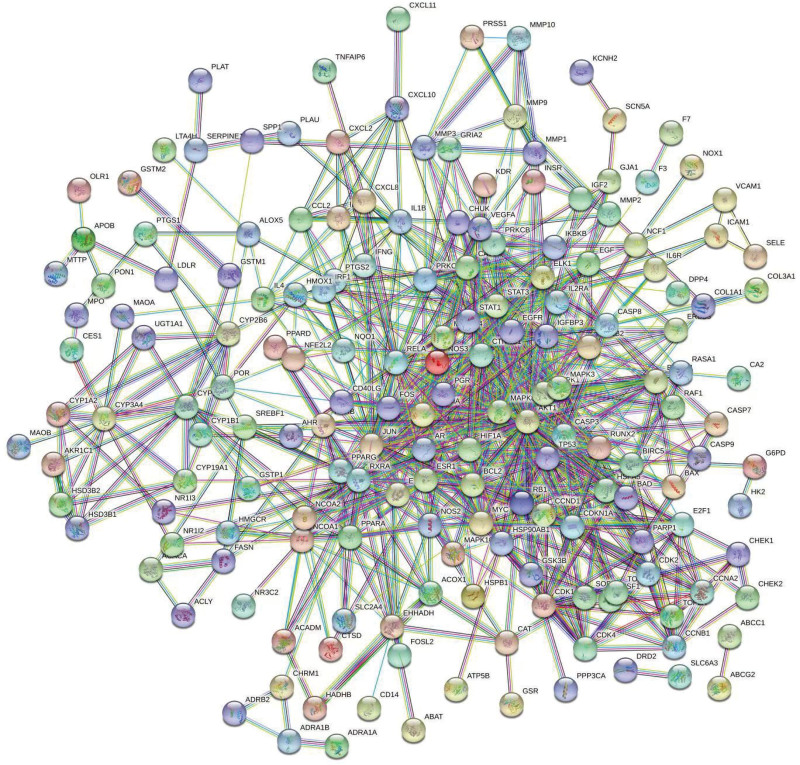
PPI network of PNI. PNI = peripheral nerve injury, PPI = protein-protein interaction.

**Figure 5.**
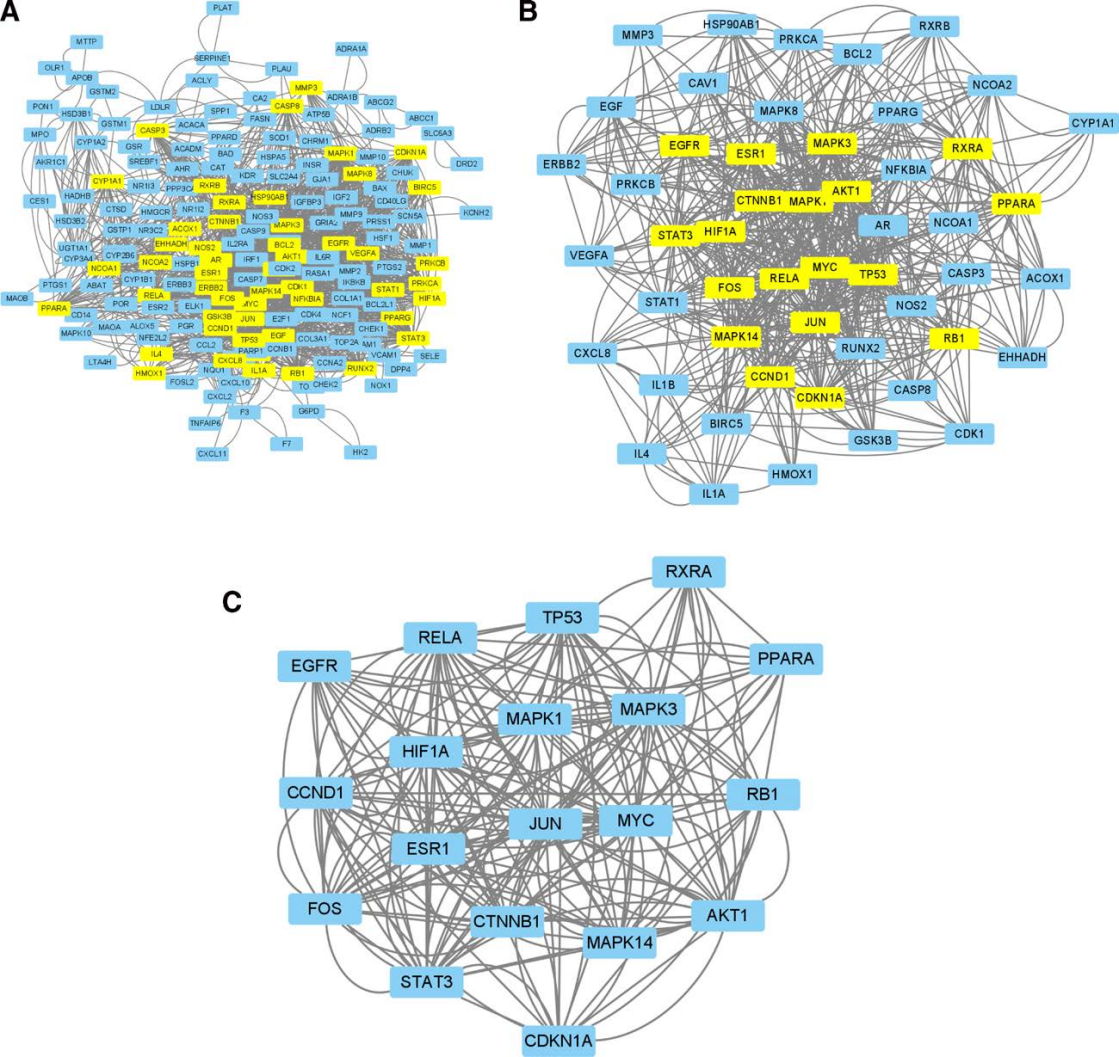
Danggui Sini decoction–PNI network topology analysis. (A) Filtered for the first time core network diagram, (B) Filtered for the second time core network diagram, and (C) Streamlined core network. PNI = peripheral nerve injury.

To further study the mechanism of the Danggui Sini decoction in the treatment of PNI, 221 intersection targets were analyzed by constructing a PPI network (Fig. 4). A total of 1554 edges representing PPI were generated to clarify the target and interaction relationship among targets. The constructed PPI network was imported into Cytoscape 3.8.2. The score of each node was calculated using the CytoNCA plug-in. Data were filtered using R, and the filter condition was that the gene score in BC, DC, CC, EC, LAC, and NC is greater than the median value. The topological network BC value was calculated as 71.21, DC value as 12, CC value as 0.14, EC value as 0.02, LAC value as 4.8, and NC value as 5.71, and we retrieved the list of genes that met the above conditions. The gene list was imported into Cytoscape 3.8.2, and the CytoNCA plug-in was used to obtain the core network, which contained 177 nodes and 1554 edges (Fig. 5A).

To simplify the network, the gene list was scored twice. The filtering condition was that the gene scores in BC, DC, CC, EC, LAC, and NC are greater than the median value. The calculated topological network BC value was 15.71, DC value was 24, CC value was 0.55, EC value was 0.103, LAC value was 10, and NC value was 11.29. Once again, the gene list was imported into Cytoscape 3.8.2, and the core network was obtained using the CytoNCA plug-in. The network contained 51 nodes and 670 edges (Fig. 5B).

A sub network was created for the obtained graph, the gene list was imported into Cytoscape 3.8.2, and the CytoNCA plug-in was used to obtain the core network, which contained 19 nodes and 395 edges (Fig. 5C). Finally, 19 core genes were obtained and are listed from high to low according to degree value: *CDKN1A, HIF1A, MYC, RXRA, PPARA, RELA, JUN, CCND1, EGFR, MAPK1, ESR1, AKT1, MAPK14, MAPK3, STAT3, RB1, TP53, FOS*, and *CTNNB1*. The genes with high connectivity with the active ingredients of drugs were selected for molecular docking analysis.

### 3.5. GO and KEGG pathway enrichment

**Figure 6.**
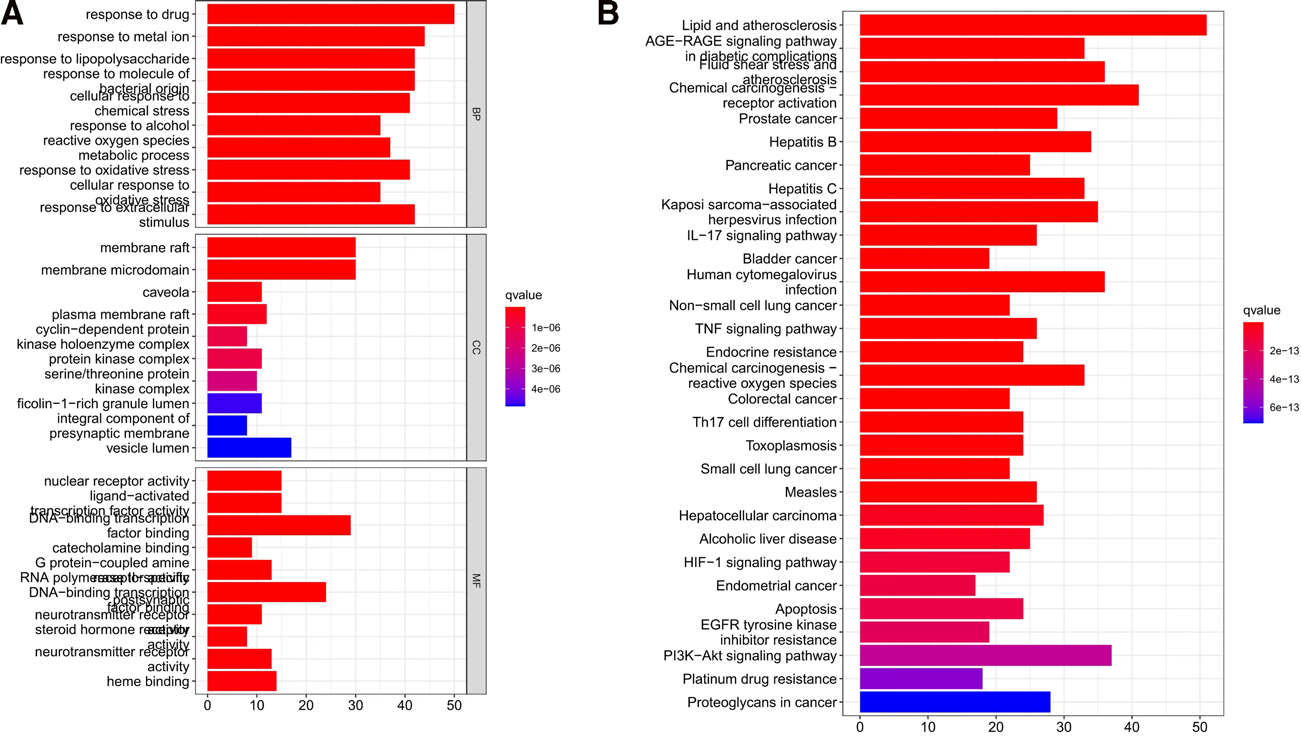
Gene enrichment at the intersection of Danggui Sini decoction–PNI. (A) GO enrichment results, and (B) KEGG enrichment results. GO = gene ontology, KEGG = Kyoto Encyclopedia of Genes and Genomes, PNI = peripheral nerve injury.

**Figure 7.**
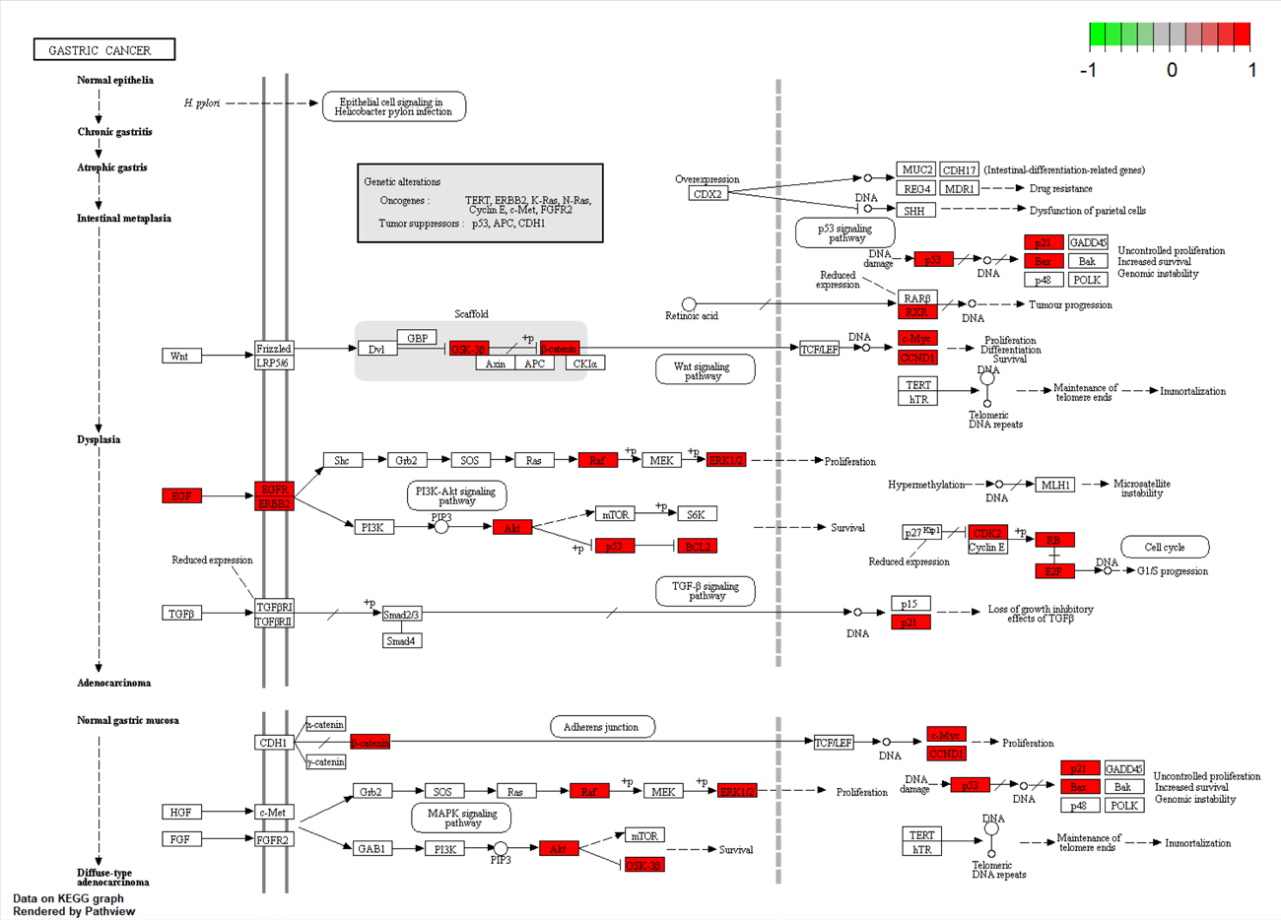
PI3K-Akt signaling pathway diagram.

A total of 2744 functions were obtained: 2410 of them were enriched for biological processes, 106 for cell composition, and 228 for molecular functions. The first 10 functions were screened and plotted (Fig. 6A). A total of 181 pathways were obtained from the KEGG pathway enrichment analysis, and the first 30 pathways were screened and plotted (Fig. 6B). There were 9 signal pathways enriched in peripheral nerve injury, namely pathways of neurogenesis – multiple diseases, lipid and atherosclerosis, AGE-RAGE signaling pathway in diabetic composites, Kaposi sarcoma associated herpesvirus infection, IL-17 signaling pathway, PI3K Akt signaling pathway, MAPK signaling pathway, fluid shear stress and atherosclerosis, and Alzheimer disease. Therefore, we suggest that the Danggui Sini decoction can act on PNI through multiple channels. Taking the PI3K Akt signaling pathway as an example, this is a potential target and mechanism of the Danggui Sini decoction in the treatment of PNI (Fig. 7). The genes marked in red in the figure are the key targets of the decoction in the treatment of PNI.

### 3.6. Molecular docking verification

**Figure 8.**
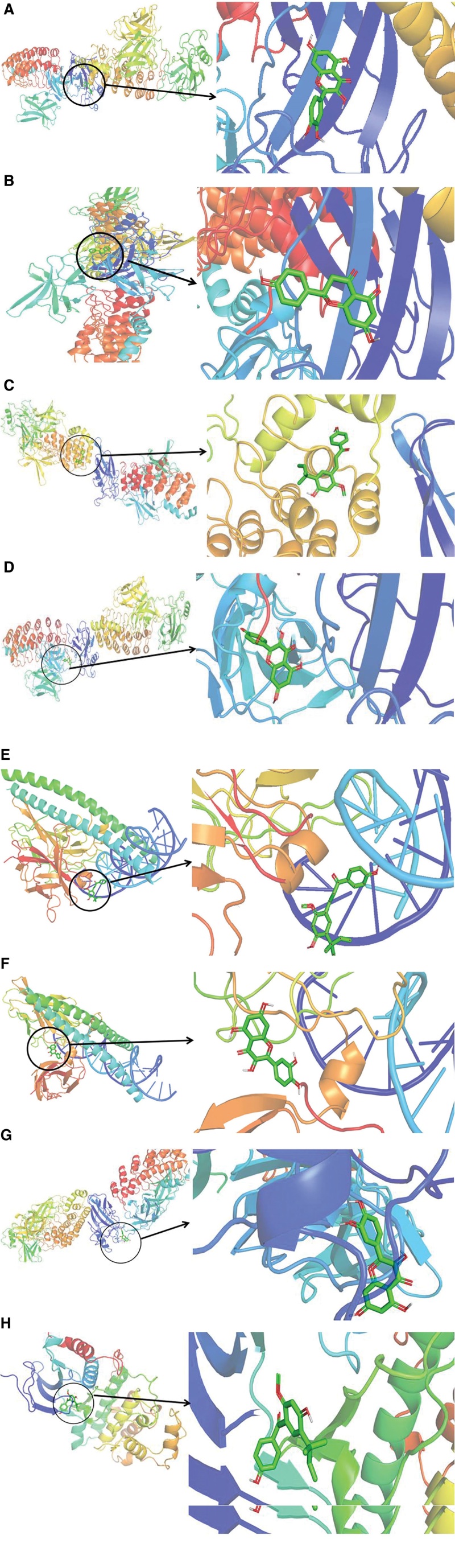
Moleculardocking results: (A) RELA-quercetin; (B) RELA-naringenin; (C) RELA-licochalcone a; (D) RELA-kaempferol; (E) FOS-quercetin; (F) FOS-licochalcone-a; (G) RB1-quercetin; (H) MAPK1-licochalcone-a; (I) MAPK1-quercetin; and (J) MAPK1-naringenin.

The core genes obtained from the network topology analysis were sorted according to degree value, and the top 4 target genes (*RELA, RB1, FOS*, and *MAPK1*) were selected for molecular docking with the 4 key pharmacodynamic components (kaempferol, quercetin, naringenin, and licochalcone A). The lower the energy when the conformation of the ligand and receptor is stable, the greater the possibility of action. The molecular docking junctions showed that the binding energy between RELA and quercetin, naringenin, licochalcone A, and kaempferolis −7.7, −7.4, −6.7, and −8.5 kcal/mol, respectively; that between FOS and quercetin and licochalcone A is −8.2 and −6.1 kcal/mol, respectively; that between RB1 and quercetin is −7.0 kcal/mol; and that between MAPK1 and licochalcone A, quercetin, and naringeninis −7.3, −8.4, and −8.4 kcal/mol, respectively (Fig. 8).

## 4. Discussion

According to the tenets of TCM, nerve injury belongs to the categories of “tendon injury” and “arthralgia,” and PNI belongs to the category of “wilting syndrome,” which can cause a range of symptoms, including muscle atrophy, weakness, and chronic pain.^[28,29]^ The pathogenesis involves vein blockage and meridian dystrophy. The Danggui Sini decoction inhibits inflammatory factors, protecting microvascular endothelial cells and relieving neuropathic pain and anticoagulation, and is commonly used to treat PNI.^[30–33]^

In this study, the TCMSP database was used to obtain the components of the Danggui Sini decoction. From this, 155 effective components and 242 potential targets were identified through network pharmacological analysis. Then, 221 genes with potential roles in the treatment of PNI by Danggui Sini were determined. We built a sub network with 221 nodes and 1554 edges in PPI. GO analysis found 2410 biological processes, 228 molecular functions, and 106 cellular components, and KEGG analysis found 181 enriched pathways. The molecular docking of the 4 targets and the 4 active components provided an in-depth analysis of genes and drug components, and we concluded that neurodegeneration, immune regulation, inflammatory response, and lipid metabolism were the most enriched functions.

The PPI network analysis showed that the core targets of Danggui Sini in the treatment of PNI included CDKN1A, HIF1A, MYC, RXRA, PPARA, RELA, JUN, CCND1, EGFR, MAPK1, ESR1, AKT1, MAPK14, MAPK3, STAT3, RB1, TP53, FOS, and CTNNB1. These targets are involved in biological processes such as neurodegeneration, inflammatory response, lipid and atherosclerosis, immune regulation, the role of AGE-RAGE signaling pathway in diabetes complications, endocrine resistance, regulation of basic cell function, cell proliferation, differentiation and migration, cell aging, apoptosis, major regulation of hypoxia induced genes, and regulation of gene transcription. Thus, this decoction can effectively reduce nerve injury after PNI and improve nerve function. Kaempferol, quercetin, naringenin, and licochalcone A were the main pharmacodynamic components docking with the targets and have been shown to have neuroprotective and neurotrophic effects. Kaempferol can achieve neuroprotective effects by reducing oxidative stress and inflammatory response.^[34]^ Quercetin improves peripheral nerve ischemia–reperfusion injury through the NF-KB pathway,^[35]^ accelerates functional recovery by upregulating the intrinsic growth ability of neurons and delaying distal atrophy,^[36]^ achieves neuroprotection by activating AMPK/PGC-1α and protecting the mitochondrial pathway,^[37]^ affecting anti-oxidative stress and promoting autophagy, which is conducive to the prevention and treatment of PNI,^[38,39]^ and showed significant analgesic and neuroprotective effects in animal models, and inhibited the formation of mild edema. Naringin can enhance nerve regeneration^[40,41]^ and both naringin and quercetin reversed the effects of hypobaric hypoxia and elicited neuroprotective responses. Licochalcone A inhibits the proinflammatory macrophage/microglia-mediated proinflammatory response, thus achieving neuroprotection.^[42]^ The results of network pharmacology highlight the potential of the Danggui Sini decoction for clinical application and its effectiveness at treating PNI.

According to the KEGG enrichment analysis, the important pathways for the treatment of PNI by Danggui Sini include neurodegeneration, inflammatory reaction, lipid and atherosclerosis, immune regulation, the role of AGE-RAGE signal pathway in diabetes complications, endocrine resistance, regulation of basic cell function, cell proliferation, differentiation and migration, cell aging, cell apoptosis, major regulation of hypoxia induced genes, and gene transcription. Research showed that kaempferol regulates pathways of neurogenesis – multiple diseases, MAPK, PI3K Akt signaling pathway, lipid and atherosclerosis, fluid shear stress and atherosclerosis, AGE-RAGE signaling pathway in diabetic composites, NF-KB, IL-17, and Alzheimer disease signaling pathway^[34,43–49]^; naringin regulates MAPK, PI3K Akt signaling pathway, NF-KB, lipid and atherosclerosis, and Alzheimer disease signaling pathway^[46,50–52]^; quercetin regulates Alzheimer disease signaling pathway, IL-17, pathways of neurogenesis - multiple diseases, MAPK, NF-KB, lipid and atherosclerosis, and AGE-RAGE signaling pathway in diabetic complications^[53–57]^; glycyrrhizin a regulates MAPK, PI3K-Akt signaling pathway, IL-17, NF-KB, and Alzheimer disease signaling pathway.^[58–61]^ The results indicate that the main components of the Danggui Sini decoction are involved in multi-level and multi-target regulation of PNI-related pathways, among which kaempferol and quercetin are involved in the most regulation pathways and the most potential targets, providing directions for the future development of clinical treatments for PNI.

Neurodegeneration and immune regulation are important factors of PNI. According to network topology and molecular docking, most of the potential targets of the main components of the Danggui Sini decoction were involved in neurodegeneration and immune regulation pathways. According to molecular docking, RELA binds more effectively to kaempferol and FOS to quercetin. Kaempferol downregulates the MAPKs-NF-KB and pyrolytic signaling pathway to reduce oxidative stress and inflammatory response.^[34]^ This improved neurological deficits in the brain of I/R rats by reducing neuroinflammation and blood–brain barrier dysfunction.^[62]^ Quercetin reduces peripheral neurotoxic lesions by inhibiting FOS through antioxidant action.^[63]^ Both RELA and FOS play roles in the c-AMP pathway. FOS and c-AMP are important regulatory pathways, which have been widely studied because they regulate metabolism, genes, neurotransmitter synthesis, growth factors, and immune function.^[64]^ c-AMP can enhance the growth and regeneration of neurites and upregulate the cAMP/PKA/CREB pathway to stimulate axonal regeneration and brain repair.^[65]^ These results combined with our results indicate that many components of our TCM act on multiple targets through multiple channels, which provides new directions for reducing neuroinflammation and regulating nerve cell growth after PNI.

## 5. Conclusions

We used network pharmacological analysis to explore the potential mechanisms underlying the action of Danggui Sini in the treatment of PNI. We found potential active ingredients (e.g., quercetin, kaempferol, isoflavones, naringenin, beta sitosterol, isorhamnetin, and stigmasterol) that regulate biological processes such as neurodegeneration, apoptosis, immune response, and lipid metabolism through molecular docking targets and enriched pathways, protect nerve cells after PNI, and prevent reinjury. This study theoretically explained the adjuvant effect of Danggui Sini on PNI. Building on the results of the present study, we aim in future work to provide biological experimental evidence for the therapeutic effect of Danggui Sini on PNI.

## Acknowledgments

We would like to thank Editage (www.editage.cn) for English language editing.

## Author contributions

**Conceptualization:** Yan Wang.

**Data curation:** Ning Zhang, Qian Zhang, Ruisu Zhang, Yan Wang.

**Formal analysis:** Ning Zhang.

**Methodology:** Dandan Zhang, Ruisu Zhang.

**Software:** Dandan Zhang, Qian Zhang.

**Writing – original draft:** Ning Zhang, Dandan Zhang, Yan Wang.

**Writing – review & editing:** Ning Zhang, Dandan Zhang.
